# Lack of significant effect of gut microbiota on weight gain in newly emerged worker honeybee

**DOI:** 10.1098/rsos.242151

**Published:** 2025-03-26

**Authors:** J. Elijah Powell, Erick V.S. Motta, Joanito Liberti, Kathleen Sotelo, Philipp Engel, Nancy A. Moran

**Affiliations:** ^1^ Department of Integrative Biology, University of Texas at Austin, Austin, TX, USA; ^2^ Department of Entomology, Texas A&M University, College Station, TX, USA; ^3^ Department of Ecology and Evolution, University of Lausanne, Lausanne, Switzerland; ^4^ Department of Fundamental Microbiology, University of Lausanne, Lausanne, Switzerland; ^5^ Department of Genetics and Evolution, University of Geneva, Geneva, Switzerland

**Keywords:** *Apis mellifera*, honeybee, microbiota, microbiome, weight gain, cuticular hydrocarbons

## Abstract

The western honeybee, *Apis mellifera,* harbours a simple and distinct microbiota that has been linked to various positive outcomes for the host. Among these cited benefits is improved weight gain for bees that have been inoculated with their native microbes. This result has been challenged by recent studies which investigated the impact of the gut microbiota on behavioural maturation and associated physiological changes and revealed no effect of the gut microbiota on weight gain. Therefore, we re-examined the role of the microbiota in weight gain by comparing microbiota-deprived bees with those inoculated with gut homogenate or defined communities composed of isolates representing the major bacterial taxa inhabiting the bee gut. We observed no differences in weight gain of adult bees or of their gut tissues across these groups. Further analysis based on nurse/forager cuticular hydrocarbon status and bacterial composition also revealed no significant changes. These results suggest the need for more nuanced investigations aimed at exploring factors such as the conditions in the hive of origin, including larval nutrition.

## 1. Introduction

The gut microbiota of honeybee workers has become an experimental model for understanding interactions within a host-specialized microbial community. This community is relatively simple, composed of only five to eight bacterial genera. The core lineages in this community include the genera *Snodgrassella* and *Gilliamella*, which colonize the ileum, and the Gram-positive *Bifidobacterium*, *Bombilactobacillus* and *Lactobacillus* nr. *melliventris* clades (previously referred to as Firm-4 and Firm-5, respectively). Other genera such as *Frischella*, *Bartonella, Commensalibacter* and *Bombella* are also frequent members of this gut community. The system is experimentally tractable as all common member species can be cultured, several genera can be genetically manipulated, and bacteria can be experimentally introduced into gnotobiotic hosts. Numerous experiments have shown effects on bee phenotypes, including some effects that appear beneficial. Inoculation with all or some of the core gut species provides protection against bacterial and eukaryotic pathogens [1–3], and disruption of the microbiota with antibiotics interferes with this protection [4–6]. Further, the microbiota include species able to break down and ferment components of the pollen husks which release short-chain fatty acids and potentially influence host metabolism [7–10].

One effect that has been reported is stimulation of insulin-like signalling, associated with elevated sensitivity to sugar and increased weight gain in young adult workers [10]. The evidence for these effects came from experiments that compared microbiota-deprived workers with workers with ‘conventional’ microbiota established by inoculation with gut homogenates derived from field-collected workers. However, another study did not find this effect and suggested that such experiments are complicated by variation in developmental trajectories of co-housed bees [11]. Specifically, within a particular co-housed group, some individuals precociously transition to a ‘forager’ phenotype, while others retain the ‘nurse’ phenotype. These individuals are expected to diverge in phenotypic features including endocrine signalling and weight gain. They also diverge in cuticular hydrocarbon (CHC) profiles, enabling the assignment of individual bees to the two developmental categories [12–14].

To better understand the generality of microbiota effects, we have re-examined the effect of microbiota presence on weight gain in newly emerged honeybee workers and have explored whether weight gain is associated with the forager versus nurse developmental switch, as detected through analysis of CHC profiles in experimental bees. Our experiments compared bees deprived of microbiota with bees with conventionalized microbiota and with bees with a defined gut community of known strains representing core microbiota species.

## 2. Material and methods

### 2.1. Rearing of gnotobiotic bees

We pulled brood frames from three research hives at the University of Texas at Austin on 5 and 6 August 2023. No permissions or licences were required to use brood from these hives. As in previous studies [15], we removed melanized dark-eyed pupae from capped brood cells with sterile forceps and placed them on Kimwipes (Kimberly-Clarke, USA) in sterile plastic bins at 35°C and approximately 60% relative humidity to replicate hive conditions. We provided irradiated pollen and sterile sucrose : water solution (1 : 1 by weight) for food as the adults emerged (approx. 150–210 bees per hive).

### 2.2. Splitting bees to experimental conditions

After the bees reached their adult morph (2–3 days), we divided the batches from each hive into three groups: (i) microbiota deprived (MD), (ii) defined community (DC), and (iii) gut homogenate (GH). For each hive, we prepared 18 cups (six cups per condition) and placed six bees in each cup (*n* = 324 bees in total, 108 from each hive and 36 per condition per hive). We marked each bee with a coloured paint mark (Sharpie paint marker, Vietnam) on the dorsal side of the thorax to track each individual bee throughout the experiment. All bees used in the experiment appeared to be normal and healthy. We did not use bees with deformed wings or abnormal abdomens. Each cup cage was provided a feeding trough (a 2 × 3 well section of a flat-bottomed culture plate (Corning, USA) filled with sterile pollen) and a 10 ml centrifuge tube (Sarstedt, Germany) used as a gravity-fed reservoir for sterile sucrose solution. The bees in MD cups were mock-inoculated by first mixing 1.5 ml sterile 1× phosphate-buffered saline (PBS) with 1.5 ml sterile sucrose solution and then placing 250 μl of this mix in the feeding trough of each cup.

### 2.3. Bacterial strain cultivation for defined community inoculations

We grew up bacterial strains (electronic supplementary material, table S1) from frozen glycerol stocks struck to Columbia Agar (BD, USA) with 5% sheep blood (CB-A). All plates and resultant liquid media were incubated at 35°C and 5% CO_2_. We allowed these plates to grow for 4 days and then used colonies from them to start 5 ml liquid cultures (electronic supplementary material, table S1 for specific media) which we incubated for 48 h. We then added 2 ml of these liquid cultures to 3 ml of fresh media and enriched these tubes overnight to obtain log phase cultures for inoculating bees. After enrichment, we measured the optical density of all cultures at 600 nm and adjusted them to a density of 1. We then combined 1 ml of each adjusted enrichment in a 15 ml Falcon tube (Corning, USA) and spun at 4°C for 15 min at 7800 r.p.m. We discarded the supernatant and resuspended the pellet in 4.5 ml 1× PBS with 4.5 ml of sterile sucrose solution. We inoculated the bees in the DC cups by adding 250 μl of this bacterial mixture to the feeding troughs.

### 2.4. Preparing gut homogenate for conventionalized inoculations

To inoculate bees in ‘gut homogenate’ cups, we gathered 20 healthy adult nurses from the inside of each of the hives from which pupae were pulled. We kept these workers in an incubator at 35°C and 60% relative humidity overnight and then dissected the guts from six bees the following day (corresponding with the date that bees were split to cups and other groups were inoculated). We transferred two guts to each of three pestle tubes and homogenized them in 500 μl of sterile 1× PBS. We combined these homogenized mixtures (1500 μl) with 1500 μl of sterile sucrose solution. We transferred 250 μl of this homogenate to the feeders in GH cups containing emerged workers from the hive of origin of the dissected bees.

### 2.5. Weighing bees

To reduce the possibility for observational bias, the technician who prepared the experimental conditions and placed bees in weighing tubes was different from the technician who performed the weighing. Measurements were blinded, so that the technician who performed weighing did not know the cup or treatment group from which the bee originated.

We prepared to weigh the bees by numbering three sets (corresponding to each hive of origin) of 2 ml microcentrifuge tubes from 1 to 108 (VWR, USA). We then obtained a blank measurement for each tube by weighing them empty on a Sartorius microbalance (Sartorius, Germany). The sample preparation technician placed each bee into the numbered tube corresponding with the numbered tube that matched its hive/cup/colour designation. The weight technician then obtained the rack of tubes containing bees and reweighed them to obtain the combined weight of the bee and tube. We calculated the individual bee weight by subtracting the blank weight from the combined weight. Any bees that we found dead were noted and discarded prior to the weighing procedure. We weighed bees at day 0 of the experiment and then on days 4, 7, 11 and 15 (electronic supplementary material, table S2).

We analysed weight change for each bee over time in a similar manner to [10], wherein we calculated the percentage of body weight gain relative to the initial body weight (set to 100) and then compared groups at each time point by using a linear mixed effects (LME) model via the R package lmerTest [16,17]. Group and time point were considered as fixed effects, and bee within cup within hive as random effects in the formula: lmer (Relative weight ~ Group × Time + (1 | Hive/Cup/Bee)). Statistical analyses were performed using the ‘Anova’ function in the R package car [18]. When comparing weight gain trends between treatment groups, we removed six samples from the MD group that had levels of bacteria at similar levels to the other groups as we believed this might skew the analysis. We performed a further evaluation based on the CHC profiles (and the assignment of nurse and forager status) for all samples retained on day 15, which is described in §2.11.

### 2.6. Weighing guts

At the conclusion of the weight gain experiment on day 15, we used sterile forceps to dissect the guts of all the live bees weighed at that time point. We placed the rectum and its contents in one labelled 2 ml tube and the carcass in another. These were immediately placed at −80°C. We left the midgut and ileum as intact units and placed them in labelled pre-weighed 0.2 ml polymerase chain reaction (PCR) tubes (VWR, USA). We maintained these tubes on ice until measurement. We weighed the dissected midgut–ileum of each individual bee by first obtaining a blank tube measurement of the PCR tubes on a Cahn 29 automatic electro balance (Cahn Instrument Company, USA). We then weighed the tubes containing these bee guts and obtained their weight by subtracting the blank weight (electronic supplementary material, table S2). After weighing, we placed these gut components at −80°C. We did not weigh the rectums as we suspected these would depend more on contents than on any developmental trend.

We compared the weight of guts in each group per hive by using an LME model via the R package lmerTest [16,17]. Group was considered as a fixed effect, and cup within hive as random effects in the formula: lmer (Gut weight ~ Group + (1 | Hive/Cup)). Statistical analyses were performed using the ‘Anova’ function in the R package car [18]. Analysis based on nurse–forager status is described in §2.11.

### 2.7. Nucleic acid extraction

We extracted nucleic acids from all *A. mellifera* gut samples by first thawing samples stored at −80°C on ice. We then combined matching dissected tissues of midgut–ileum dissections and retained rectal tissues by placing them in clean pestle tubes with 600 μl of RNA lysis buffer from the Zymo Quick RNA Miniprep kit (Zymo, USA). We homogenized the abdomens for 30 s with a pestle (Biospec, USA) and then placed this mixture into bead tubes containing 500 μl of 0.1 mm silica zirconia beads (Biospec, USA). We then bead beat the samples for 1 min, allowed them to cool on ice for 30 s, and bead beat them again for 1 min. We then used the Zymo kit as per the manufacturer’s instructions (including the optional in-column DNase treatment) to prepare 100 μl of RNA. We also isolated DNA from these same samples by retaining the gDNA elimination column from the RNA preparations. We washed these DNA columns with 500 μl of Qiagen wash 1 and 2 buffers from the Qiagen Dneasy kit (Qiagen, USA), spun them at 14 000*g* for 2 min and then eluted the DNA with 100 μl of molecular grade water. We stored RNA at −80°C and DNA at −20°C.

### 2.8. 16S rRNA gene metabarcoding

For bacterial community surveys based on 16S rRNA gene V4 metabarcoded amplicons, we constructed sequencing libraries in a similar fashion to that used in previous studies from our laboratory [5,19,20].

We used DNA from the combined gut samples of the weighed midguts–ileum and retained rectal tissue from all samples weighed at the end of the weight experiment. We conducted the first PCR reaction using 5 μl of DNA template in 20 μl reactions with Illumina-adapted 515F and 806R primers [21] using Accustart 2 mastermix (Quanta Bio, USA). Cycling conditions, primers and mastermix recipes may be viewed in electronic supplementary material, table S3). We examined these reactions on a 2% agarose gel, then purified products with 0.8× HighPrep PCR magnetic beads (MAGBIO, USA). The cleaned product was diluted to a final volume of 52.5 μl. We performed the second PCR reaction to attach Illumina 8 bp Nextera style dual-indexed barcodes to 1 μl of the PCR 1 product in 25 μl single reactions using a unique combination of N7XX and S5XX primers. We cleaned these reactions with magnetic beads, resuspended in 27.5 μl molecular grade water and quantified them on a plate reader using the Accublue broad range dsDNA quantitation kit (Biotium, USA). We pooled equimolar amounts of each amplification, combined with 7% PhiX DNA and sequenced on an Illumina MiSeq instrument at 2 × 250 reads (Illumina Corp., USA).

The V4 metabarcode reads were demultiplexed onboard the iSeq (Local Run Manager, Generate FASTQ Analysis Module 2.0). We then processed the forward reads with QIIME 2 version 2023.9 [22]. We removed primer and adapter sequences with the cutadapt plugin [23] and truncated to 120 base pairs. We filtered, denoised and removed chimeric reads using the Deblur plugin [24]. We assigned taxonomic classifications to amplicon sequence variants (ASVs) using the SILVA 132-99-515-806-nb database with the feature-classifier plugin [25]. We inspected ASVs and removed unassigned, mitochondrial, chloroplast and singleton reads with the feature-table plugin [26]. We used the align-to-tree-mafft-fasttree phylogenetic tree building function [27] to reconstruct a phylogenetic tree to use with diversity estimation models. We visualized the relative taxonomic composition of bee gut communities with the taxa-barplot command within Qiime2 [26].

We examined alpha diversity within samples by performing 10 subsampling iterations at every 100 reads at each sampling interval. We used a uniform depth of 9500 reads per sample, as this depth preserved a high number of samples for examination and showed saturation of alpha diversity. We examined species richness by obtaining the Shannon index for each sample. We compared this diversity metric between groups using the linear mixed model and ANOVA strategy noted previously. Following this, we compared multiple groups within the model using emmeans [28]. We plotted graphs in R using ggplot2 [29]. We compared beta diversity between groups by using weighted UniFrac distance matrices and testing for significance with PERMANOVA tests [30]. We plotted principal coordinate analysis (PCoA) ordinations within Qiime2 and used analysis of composition of microbiomes (ANCOM) to analyse differential taxonomic relative abundances [31]. The sample-related ASV table and alpha diversity information can be found in electronic supplementary material, table S4.

### 2.9. 16S rRNA gene absolute abundance

We assessed total 16S rRNA gene copy number by using quantitative PCR (qPCR) to quantify absolute numbers of copies in 1 μl of 1 : 10 diluted template of isolated gut DNA using previously described methods [15]. Briefly, we used dilutions of a defined concentration of the plasmid pGemT (Promega, USA) bearing the target segment of the 16S rRNA gene as a standard curve. We used the primers 27F/355R [32] to amplify this sequence in triplicate reactions of all samples and used the standard curve to obtain the copy number concentration. Samples that came up at or below the 1 × 10^3^ standards (the low end of the dynamic range of this assay) were rerun using the undiluted samples. We corrected resultant counts for dilution and obtained estimates for the total bacterial concentration per gut (electronic supplementary material, table S2). We created boxplots of total 16S rRNA gene copies from each experimental condition per hive and compared the total amounts between experimental conditions using the linear mixed effects models (lmer(Copies of 16S rRNA gene ~ Group + (1 | Hive/Cup)) and the interactions between these conditions and hives of origin (lmer(Copies of 16S rRNA gene ~ Group × Hive + (1 | Cup)). We ran ANOVA with these models and further investigated pairwise interactions with emmeans.

### 2.10. Obtaining cuticular hydrocarbon data

To test whether the transition from nurse to forager within these tested bees contributed to weight change, we obtained CHC data in the manner previously described [11]. We submerged the abdomen and thorax of each dissected carcass from the end of the weight gain experiment (*n* = 219 bees) in pure hexane for 10 min. We then allowed the resultant extracts to reduce via evaporation to 100 μl. We ran these extracts on an Agilent 8890-5977B GC-MS (Agilent Technologies, USA) at the University of Lausanne by ramping the oven from 65°C to 215°C at 25°C min^−1^ and then to 300°C at 8°C min^−1^. Data were acquired and processed with the ChemStation software v. F.01.03.2357 (Agilent Technologies, USA). Identification of the compounds was accomplished by comparison of library data (NIST 51420) with mass spectral data of commercially purchased standards for *n*-alkanes (Supelco, EC no. 203-625-9), diagnostic ions and retention indices.

### 2.11. Analysis of cuticular hydrocarbon data

We obtained CHC profile data for all the bees (*n* = 219) that had weight gain and qPCR data. We used the ChemStation software to calculate the relative abundance of CHC compounds. To do this, the area under each compound peak on the GC was quantified through integration and divided by the total area under all CHC peaks. The resulting raw data were aligned using the R package GCalignR v. 1.0.5 [33]. Areas under the peak values were converted to relative proportions. We then removed compounds that were not present in at least half the samples of one treatment or were only present at less than 0.1% relative abundance in all samples. We used data from previous studies [11] to construct a manually curated database and matched profile data to discard non-CHC compounds. The final dataset included 36 compounds (electronic supplementary material, table S5). We calculated Bray–Curtis dissimilarities between samples and performed non-metric multi-dimensional scaling (NMDS) ordination analyses and permutational multivariate analysis of variance (PERMANOVA) with 999 permutations (*Adonis2* function) to assess differences between experimental groups using the package vegan v. 2.6-4. Next, we calculated the multivariate centroids from each cage using the *Betadisper* function (package vegan) to account for sampling multiple individuals from the same cages and tested for the effect of gut microbiota treatment on CHC profiles using the resulting matrix. To classify bees as nurses or foragers, we used the *hclust* function of the base R package ‘stats’ to perform hierarchical cluster analyses of Euclidean distances between CHC profiles using the Ward’s criterion. To assess the effect of gut microbiota treatment on the proportion of CHC-classified nurses and foragers, we used generalized linear mixed models fitted by maximum likelihood using a binomial distribution, adding hive and cage as nested random effects to the models to account for sampling multiple individuals from the same cages.

To examine trends in weight gain based on nurse–forager status, we used the linear mixed effects model: lmer(WeightGain ~ Day + CHC classification + (1|Hive/Cage/Sample_ID)) (the interaction between Day and CHC classification was not significant) and examined the model via ANOVA. For examining proportions of nurses and foragers in hives and inoculation groups, we used a binomial structure for the generalized linear effects model: glmer(CHC classification ~ Treatment + (1|Hive/Cage)). For the endpoint test of gut weight between bees binned as nurses and foragers, we used a *t*‐test.

## 3. Results

### 3.1. There was no detectable effect of microbiota treatment on weight gain

**Figure 1.**
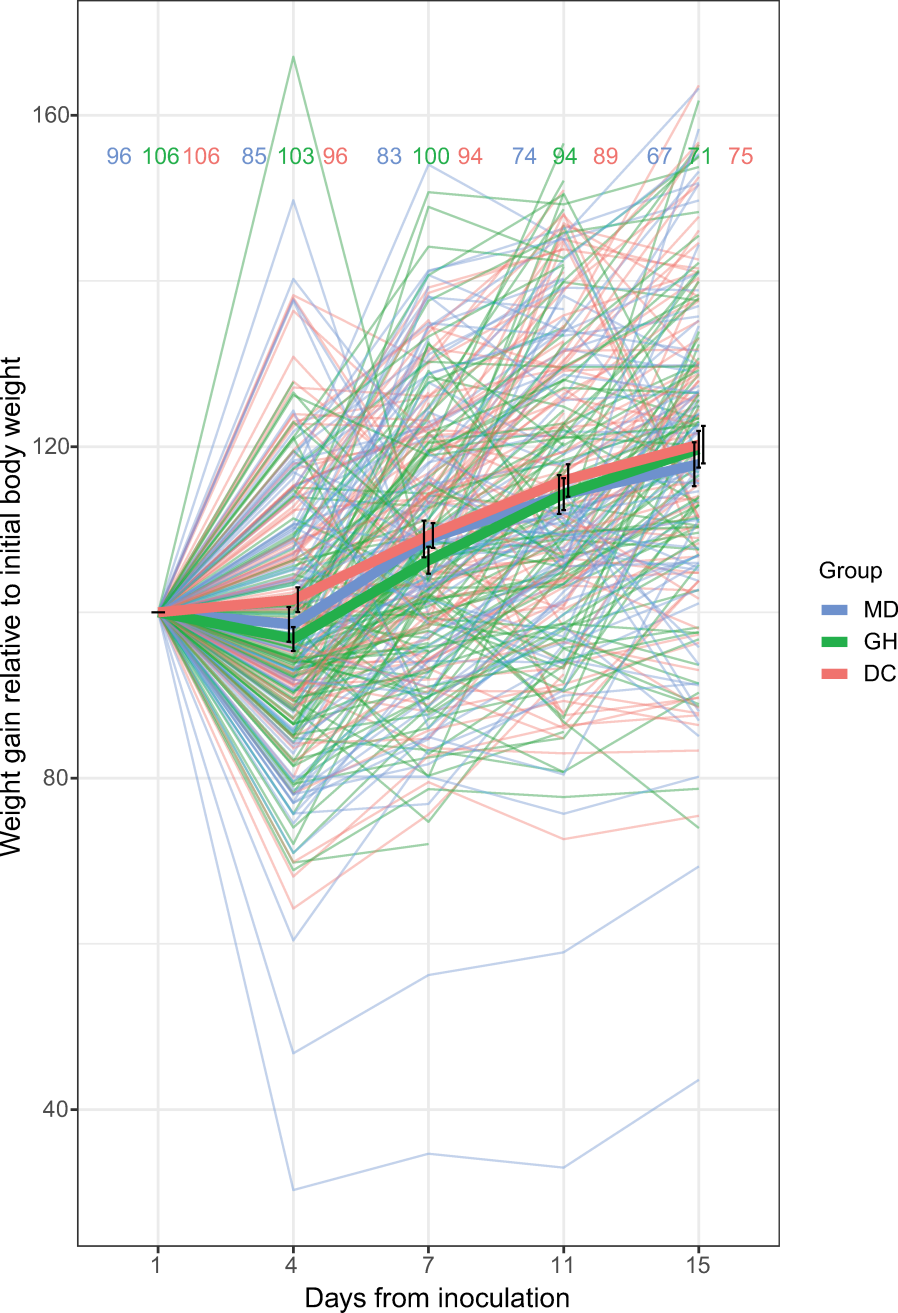
Overall weight gain. Whole body weight change relative to initial body weight for all samples in the microbiota deprived (MD), gut homogenate fed (GH) and defined community fed (DC) groups. All groups gained weight equivalently (*p* > 0.05, ANOVA test, LME model).

Bees in the different treatments gained weight at a comparable rate. Pooling all bees across hives and treatments, the average absolute weight was 0.09516 g on day 0 and 0.11136 g on day 15, showing a 17.0% increase. When examining the complete dataset, weights for all treatments were significantly different from day 0 by days 7, 11 and 15 (figure 1) (ANOVA test, LME model; χ^2^ = 591.39, d.f. = 4, *p* < 2 × 10^−16^, *n* = 1339), but treatment groups (ANOVA test, LME model; χ^2^ = 1.61, d.f. = 2, *p* = 0.45, *n* = 1339) or hive (ANOVA test, LME model; χ^2^ = 2.13, d.f = 2, *p* = 0.35, *n* = 1339) were not.

**Figure 2.**
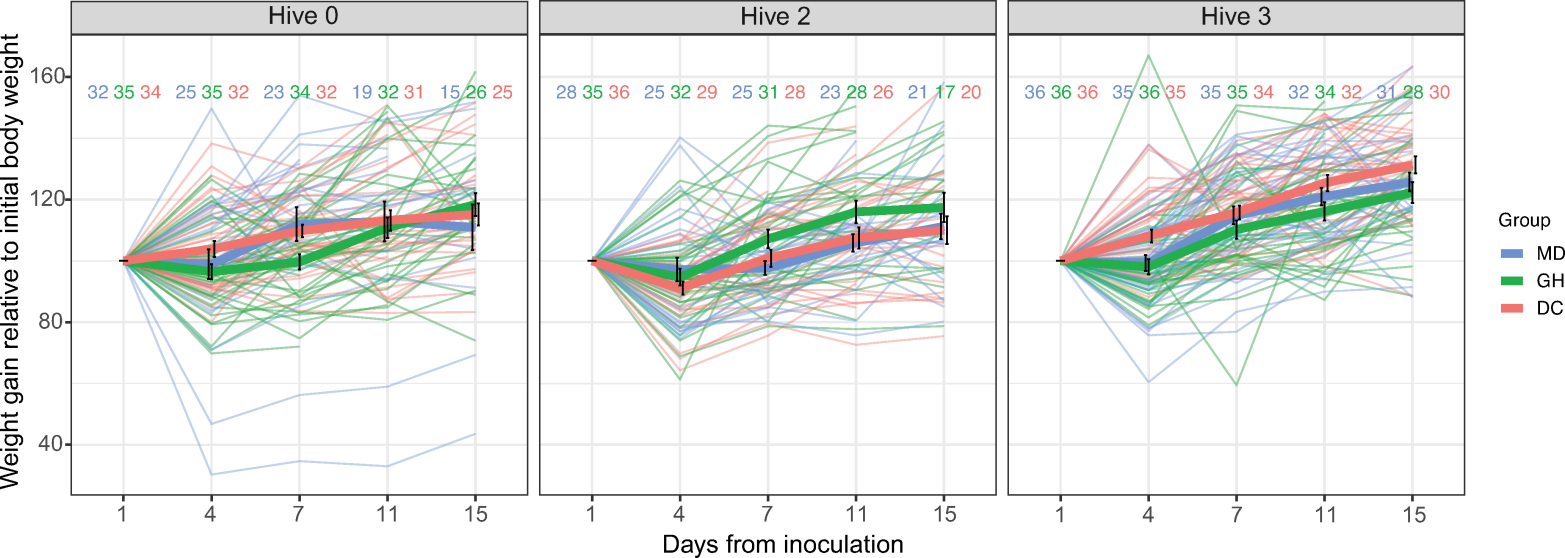
Hive weight gain. Final body weight relative to initial body weight for inoculation groups by hive. All groups gained weight equivalently within each hive (*p* > 0.05, ANOVA test, LME model).

These observed trends were similar at the hive level, though there were some small differences on given days (figure 2). These day-to-day differences did not provide clear evidence for differences between experimental groups for each hive’s bees.

### 3.2. Final weights of dissected midguts and ilea did not differ by treatment

**Figure 3.**
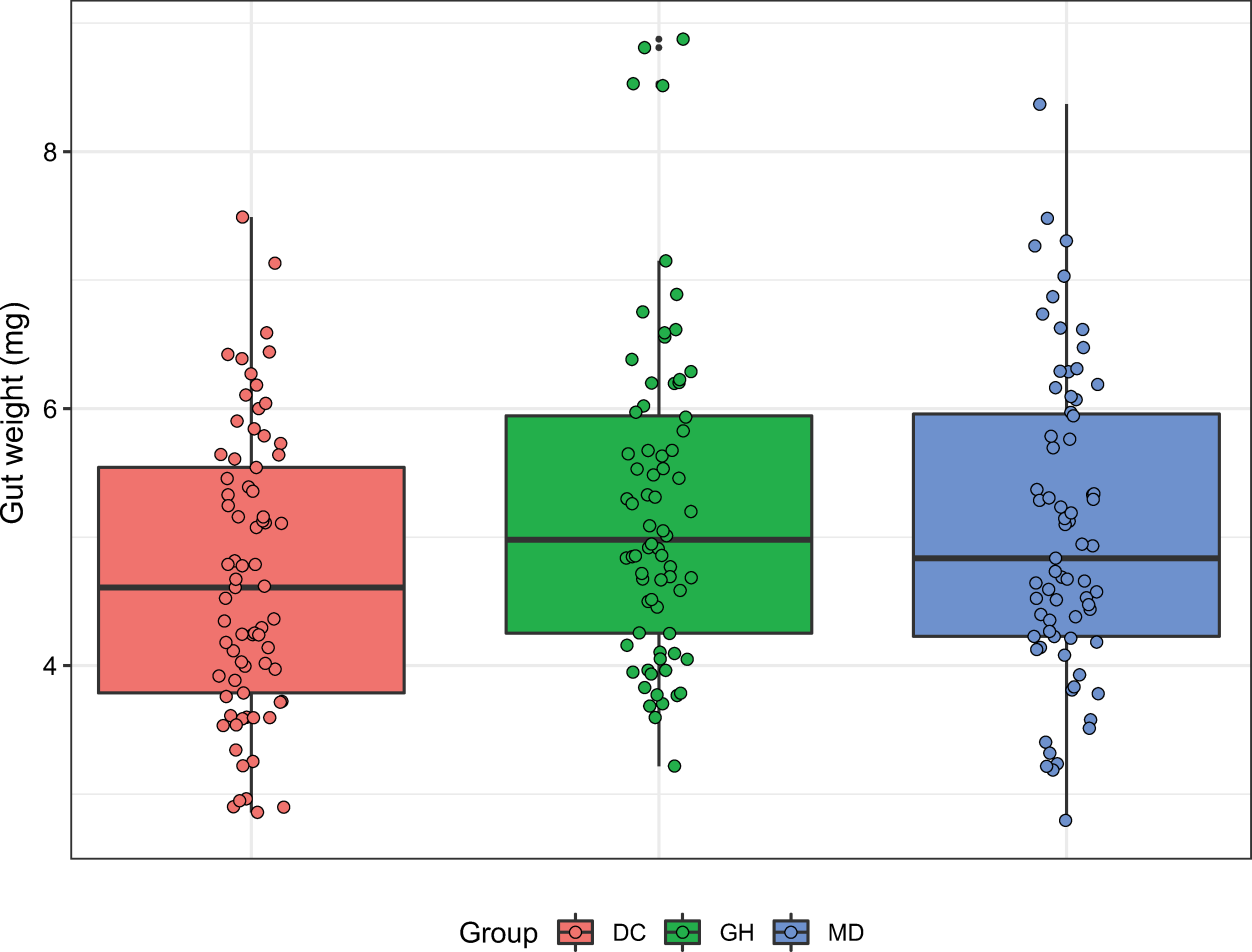
Midgut–ileum weight. Endpoint weight of midgut–ileum regions by condition. Weights were statistically equivalent (*p >* 0.05, emmeans multiple comparisons after ANOVA, LME model).

For the dissected midgut–ileum from the final timepoint (day 15), we did not observe significant differences between the groups. The average weight was 4.99 mg ± 0.08 mg (s.e.m.) for all samples (figure 3).

### 3.3. Quantitative polymerase chain reaction estimates of bacterial loads verified inoculation status

As expected, the total 16S rRNA gene copy number was low in microbiota-deprived bees (approx. 4 × 10^3^ copies per gut ± 0.2 s.e.m.), whereas it was high and statistically similar in bees that were inoculated with either a conventional gut microbiota or defined community (approx. 6.7 × 10^8^ copies per gut ± 0.06 s.e.m.) (electronic supplementary material, figure S1). This difference in load was the case regardless of the hive of origin.

### 3.4. Metabarcoding-based weighed UniFrac principal coordinate analysis results demonstrated clustering by inoculation condition (defined community versus gut homogenate) and clustering by hive of origin for gut homogenate bees

**Figure 4.**
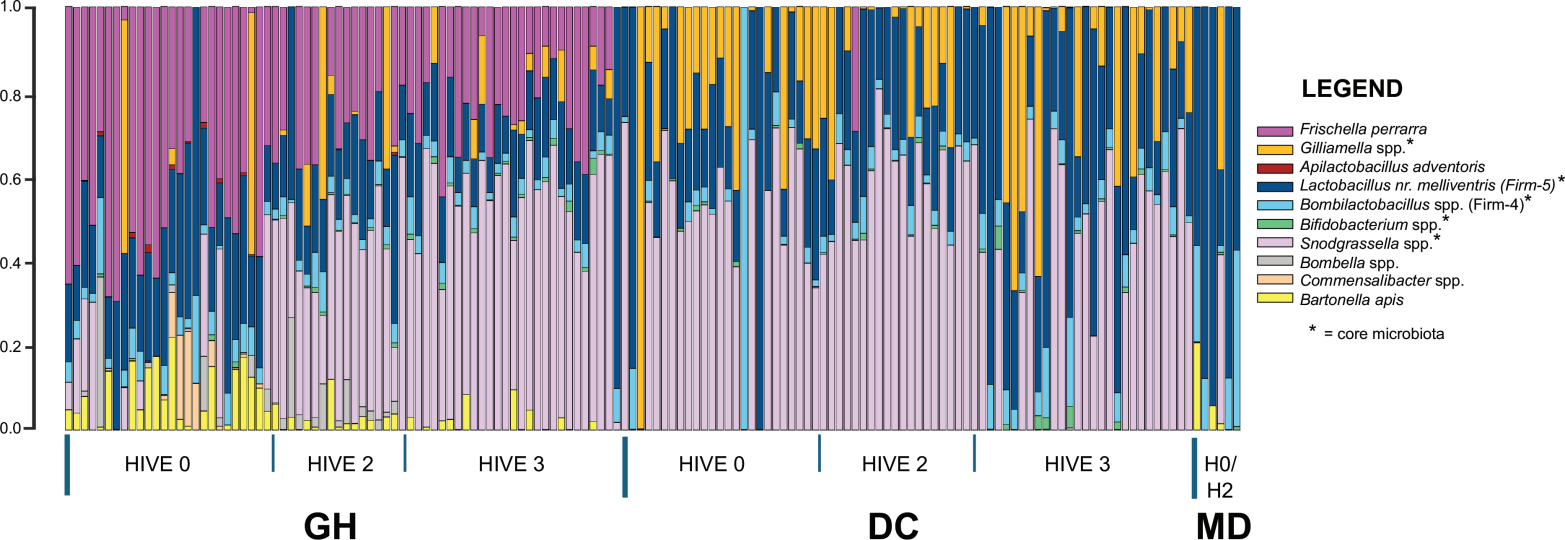
Taxonomic barplots. Stacked barplots of the relative abundance of taxonomic lineages in each sample organized by inoculation status and hive of origin. Proportions are based on 16S rRNA gene ASV read data.

Only a few MD bees amplified with the 16S rRNA gene V4 primers and had sufficient reads for analysis after rarefaction (*n* = 7 of 73 MD samples that had nucleic acids extracted at the end of the experiment), and so observations are most pertinent to DC and GH groups. The overall taxonomic composition of the samples is shown in figure 4. As compared with DC bees, the GH bees contain high relative abundances of *Frischella perrarra* and *Bartonella apis* and low abundances of *Gilliamella* spp. (and *Snodgrassella* spp. for hive 0). These differences are reflected in the ANCOM analysis, which indicated these species along with *Bombella* spp. as drivers in clustering by condition (all taxa W = 14, ANCOM). PCoA reveals clear clustering by inoculation condition for the ordinations within each hive (electronic supplementary material, figure S2 A–C), as well as for the whole dataset (electronic supplementary material, figure S2D). For GH samples alone, there were distinct clusters by hive (electronic supplementary material, figure S2E) (PERMANOVA, pseudo-*F* = 16.73; *p* = 0.001). Based on differential abundance analysis for GH samples using ANCOM, differences in *Snodgrassella* (*W* = 14), *Commensalibacter* (*W* = 14) and *Bombella* (*W* = 14) abundances were the largest drivers contributing to these differences.

### 3.5. Cuticular hydrocarbon profiles and proportions of nurses and foragers differed within groups of bees originating from a given hive but not by microbiota status

We performed hierarchical clustering of CHC profiles to classify bees into nurses and foragers. While CHC profiles differed between bees sampled at different time points, there was no effect of microbiota treatments on the CHC profile (permutational multivariate analysis of variance using Bray–Curtis dissimilarities calculated from the centroids of each cage: *n* = 53, time, *F*_2,52_ = 3.81, *R*^2^ = 0.13, *p* = 0.001, treatment, *F*_2,52_ = 1.16, *R*^2^ = 0.04, *p* = 0.14), consistent with recent findings [11]. In total, we classified 130 bees as foragers and 89 as nurses (electronic supplementary material, figure S3). In each of the three groups (MD, GH, DC), the proportion of foragers averaged between 53% and 68% (electronic supplementary material, figure S4A). When examining by hive, there are large differences between each with the proportions of foragers in all groups in hive 0 at 91%, hive 2 at 20% and hive 3 at 65% (electronic supplementary material, figure S4B). These trends are further visualized in the principal component analysis (PCA) ordinations of CHC profiles where clustering by nurse/forager status is observed by hive but not by inoculation condition (electronic supplementary material, S5).

### 3.6. The weight gain trend of nurses was not different from that of foragers

CHC-classified foragers had similar weight gains to nurses overall (ANOVA test, LME model; *F*_1,203_ = 2.41, *p* = 0.12), despite a trend towards higher weight gain in nurses in two out of three hives (electronic supplementary material, figure S6). We do not know at what point in their development they transitioned, as we were only able to determine their status at the end of the experiment.

### 3.7. The endpoint weight of the midgut–ileum was not different for nurses and foragers

The weights of the midgut–ileum compartments were statistically similar for both nurses and foragers for the whole dataset (electronic supplementary material, figure S7).

## 4. Discussion

The results of this study showed different weight gain trends compared with the findings of Zheng *et al.* [10]. That paper reported significant differences in weight gain and the weight of gut compartments between MD (labelled microbiota-free in that paper) and GH groups starting from day 7, with GH bees gaining more weight. In contrast, we found no significant differences in weight gain in any of the groups, including bees fed cultured isolates in the DC group (introduced to minimize the impact of pathogen transmission associated with GH inoculations). This finding is consistent with a recent study that also did not reveal any consistent differences in weight gain between microbiota-deprived and colonized bees, across several independent experiments and using different dietary treatments [11].

Despite variation in the microbiota presence and composition among the groups (near-total absence in MD, minimal core microbiota in DC and complex communities in GH), all groups showed similar weight gain over time.

Differences in results across studies could stem from several factors. We consider these here with the goal of improving how investigations of bee microbiota are conducted and interpreted.

### 4.1. Differences in strain and species composition can affect outcomes

Zheng *et al*. [10], Liberti *et al*. [11] and the current study used inocula consisting of complex communities taken directly from hosts, and these communities vary in composition. In our study, GH samples exhibited unusually high abundances of *Frischella perrarra* and *Bartonella apis*, potentially affecting host responses, as *F. perrara* has genotoxic properties [34], and *B. apis* shares evolutionary traits with mammalian pathogens [35]. One solution is to use inocula consisting of a defined community, consisting of strains shared among research groups. However, we note that our DC inoculum (which lacked *F. perrarra* or *B. apis*) gave results similar to those with the GH inoculum. More generally, we note that different strains within single gut microbiota species have different gene sets, including genes that enable utilization of dietary components [7–10], so it might be expected that they will differ in effects on hosts.

### 4.2. Host genotype

Host genotype could be a factor in responses to gut communities, as suggested by one study [36]. Host genotypes were not controlled across Liberti *et al*. [11], Zheng *et al*. [10] and the current study, and their influence on bee responses to microbiota is unknown.

### 4.3. Quality of the adult diet during the course of the experiment may affect interactions with gut bacteria

Adult workers are maintained on sucrose solution and pollen during experiments, but the compositions have varied across studies. Zheng *et al*. [10] used 0.5 M sucrose solution, whereas the current study and Liberti *et al*. [11] used substantially higher concentrations (1 : 1 w/v or approx. 2.9 M and 1 : 2 w/v or approx. 1.5 M, respectively). Floral nectars vary widely in composition and concentration, but are mainly composed of sucrose (a disaccharide) plus glucose and fructose (hexose monosaccharides) [37]. Typical bulk sugar concentrations of bee-visited agricultural crop species average around 40% by weight, while surveys of all genera visited by bees report concentrations of 6.3%–85% [38]. With these disparate conditions in mind, we might speculate that a lower concentration of available sugar, as in the Zheng *et al*., experiments could increase caloric need and thereby cause bees that were stimulated by microbial presence to increase syrup consumption. In the current study, a higher concentration of sugar might have enabled base caloric needs to be met more easily, potentially limiting responsiveness to gut colonization.

Pollen quality may also have an impact. A study that compared microbiota of honeybees from colonies fed on fresh versus aged pollen showed that the aged pollen diet resulted in impaired development, reduced weight, reduced adult survival and marked changes in gut community composition [39]. Specifically, feeding on aged pollen resulted in an elevated abundance of *F. perrara*, similar to the levels observed in our study and consistent with the possibility that nutritional stress in the drought-exposed colonies was a factor in our experiments. In another study, *B. apis* was more abundant in bees fed poor-quality pollen than in bees fed high-quality pollen [40]. The multi-floral pollen used in this study and in Zheng *et al*. [10] was sourced from a major supplier and irradiated prior to long-term freezing. The length of pre-sale storage and nutritional composition of this source is unknown. Investigations into microbial impacts on host development and nutrition could benefit from standardization of pollen source and treatment.

### 4.4. The larval environment is an underappreciated factor affecting studies of gut communities in adult worker bees

In almost all studies, experimental bees are sourced from outdoor hives. While effects of larval conditions can be randomized within a single study, results might not be generalizable across studies as hive conditions vary widely. Seasonality, nutritional stress and other factors affect developing larvae, and numerous studies show that larval conditions affect adult worker phenotypes, such as survivorship, feeding behaviour and development [41–43].

Season and larval nutrition differed substantially between our study and that of Zheng *et al*. Hives were at the same location, but the Zheng *et al*. experiments were performed during spring 2016, when flowers were abundant, whereas the current study was carried out in autumn 2023, following an abnormally hot and dry summer and consequent paucity of floral resources (https://www.ncdc.noaa.gov/). One likely indicator of the harsh conditions in our experiment is the relatively low absolute weights of the worker bees on both day 0 and day 15, at about 0.1 g, whereas averages in other studies have been somewhat higher [44,45].

### 4.5. Behavioural status of adult workers may confound effects of microbiota

Liberti *et al*. [11] found that weight gain was associated with nurse versus forager status, whereas our current study did not find significant differences. However, we did observe a trend of lighter weights for foragers in two out of three hives, which aligns with the Liberti *et al*. study. This apparent discrepancy should be interpreted cautiously as there were skewed distributions of foragers across hives and cages in our experiment, and the CHC status was only interpreted at the end of the experiment (day 15). While proportions of nurses and foragers varied within groups, they did not differ by microbiota status, consistent with Liberti *et al*. [11].

As for honeybees, studies on *Drosophila melanogaster* have also varied in conclusions about how gut microbiota affects metabolism and weight gain. In some studies, significant increases in adult lipid storage and body weight are linked to the presence of a gut community [46,47]. However, effects can vary and are influenced by host genotype [48], community composition [49] and diet [50].

Many questions remain concerning how bee genetics, gut community composition and larval conditions affect honeybee metabolism and development. Future experiments elucidating these factors are essential to uncover the effects of the bee gut microbiota on developmental and metabolic outcomes.

## Data Availability

All data used to create tables and figures is available in the supplementary data files [51]. 16S rRNA metabarcode sequence data from individual bees is available in Bioproject PRJNA1119460.
